# Prevention and control of non-communicable diseases in iran: the case for Investment

**DOI:** 10.1186/s12889-022-13615-w

**Published:** 2022-06-24

**Authors:** Mahmood Yousefi, Ilker Dastan, Farbod Alinezhad, Mansour Ranjbar, Christoph Hamelmann, Afshin Ostovar, Alireza Moghisi, Sima Mohammadi, Awad Mataria, Asmus Hammerich, Slim Slama, Nasim Pourghazian, Alireza Mahdavi Hezaveh, Behzad Valizadeh, Parisa Torabi, Mehdi Najmi, Mohammad Moradi, Alieh Hodjatzadeh, Fatemeh Keshvari-Shad

**Affiliations:** 1 Department of Health Economics, School of Management and Medical Informatics, Tabriz University of Medical Sciences, Tabriz, Iran; 2 Health Policy, WHO Country Office, Dushanbe, Tajikistan; 3 Bouve Colleage of Health Sciences, Northeastern University, Boston, MA USA; 4 National Professional Officer, NCD and Mental Health Unit Head, WHO , Tehran, Iran; 5 World Health Organization Representative in I.R.Iran, WHO, Tehran, Iran; 6 Osteoporosis Research Center, Endocrinology and Metabolism Clinical Sciences Institute, Tehran University of Medical Sciences, Tehran, Iran; 7 Deputy General Director for NCD Management Office, Ministry of Health and Medical Education, Tehran, IR Iran; 8 Islamic Azad University, Tehran, Iran; 9 Universal Health Coverage/Health Systems (UHS), World health Organization, Regional Office for Eastern Mediterranean (WHO-EMRO), Cairo, Egypt; 10 UHC/NCDs, World Health Organization, Regional Office for Eastern Mediterranean (WHO-EMRO), Cairo, Egypt; 11 Non Communicable Diseases Prevention (NCP), UHC/NCDs, World Health Organization, Regional Office for Eastern Mediterranean (WHO-EMRO), Cairo, Egypt; 12 Non Communicable Diseases Prevention (NCP), UHC/NCDs, World Health Organization, Regional Office for Eastern Mediterranean (WHO-EMRO), Cairo, Egypt; 13 Cardio Vascular Diseases Prevention Department, NCD Management Office, Ministry of Health and Medical Education, Tehran, IR Iran; 14 National Tobacco Control Secretariat, Ministry of Health and Medical Education, Tehran, IR Iran; 15 National Manager of Clinical Nutrition Group, Ministry of Health and Medical Education, Tehran, IR Iran; 16 Director of Respiratory Diseases Prevention Department, NCD Management Office, Ministry of Health and Medical Education, Tehran, IR Iran; 17 Expert of Cardio Vascular Diseases Prevention Department, NCD Management Office, Ministry of Health and Medical Education, Tehran, IR Iran

**Keywords:** Return on investment, Non-communicable diseases, Policy intervention, Clinical intervention, Cost; burden

## Abstract

**Background:**

Non-communicable diseases are imposing a considerable burden on Iran. This study aims to assess the Return on Investment (ROI) for implementation of Non-communicable diseases (NCDs) prevention program in Iran.

**Methods:**

Four disease groups including cardiovascular diseases, diabetes, cancer, and respiratory diseases were included in our ROI analysis. The study followed four steps: 1) Estimating the total economic burden of NCDs using the Cost-of-Illness approach. 2) Estimating the total costs of implementing clinical and preventive interventions using an ingredient based costing at delivering level and a program costing method at central level.3) Calculating health impacts and economic benefits of interventions using the impact measures of avoided incidence, avoided mortality, healthy life years (HLYs) gained, and avoided direct treatment costs. 4) Calculating the ROI for each intervention in 5- and 15- year time horizons.

**Results:**

The total economic burden of NCDs to the Iranian economy was IRR 838.49 trillion per year (2018), which was equivalent to 5% of the country’s annual Gross Domestic Product (GDP). The package of NCD will lead to 549 000 deaths averted and 2 370 000 healthy life years gained over 15 years, and, financially, Iranian economy will gain IRR 542.22 trillion over 15 years. The highest ROI was observed for the package of physical activity interventions, followed by the interventions addressing salt, tobacco package and clinical interventions. Conclusions

NCDs in Iran are causing a surge in health care costs and are contributing to reduced productivity. Those actions to prevent NCDs in Iran, as well as yielding to a notable health impact, are giving a good economic return to the society. This study underscores an essential need for establishment of a national multi-sectorial NCD coordination mechanism to bring together and strengthen existing cross-agency initiatives on NCDs.

## Background

Accounting for 42 million deaths worldwide (74% of all deaths) during 2019, non-communicable diseases (NCDs) were the leading cause of mortality globally. 61% of these deaths occur prematurely in the population below the age of 70. These proportions have seen an ever-increasing trend in recent years, especially with the urbanization of the societies in the low- and middle-income countries (LMICs) [1–3].

Iran, a lower-middle-income country, is not an exception in this regard. 83% of deaths in the country occur due to NCDs [1, 4]. The country faces an increase in its elderly population, a trend not seeming to decline in the near future, signaling a pressing need for provident planning [5–8]. Of the ten leading causes of mortality in the country, eight are classified as NCDs, ischemic heart diseases being the most prevalent, seeing an increase of 29.9% in the last ten years. Furthermore, most of the leading risk factors causing deaths and disabilities in Iran are either behavioral risk factors for NCDs, including tobacco use and dietary risks, or intermediate-risk factors, including high blood pressure, high body-mass index, high fasting plasma glucose, and abnormal lipid profiles[1].

Based on these alarming figures, target 3.4 of the United Nations Sustainable Development Goals aims to reduce the premature mortality from the NCDs to one third by 2030[9]. A critical issue regarding the increasing prevalence of the NCDs in different populations is the economic losses they lead to, both directly, through increasing the healthcare expenditures in these populations, and indirectly, through the productivity loss due to loss of working-age population as well as decreased efficiency of the population living with these conditions. The harms caused by these losses are not isolated to the healthcare sector. By decreasing the number of funds available to the countries, especially LMICs, many other aspects of development such as education, poverty reduction, gender equality, and environmental efforts may face substantial challenges. It is estimated that in the 20 years from 2011 to 2030, there will be a loss of 46.7 trillion dollars globally, most of which will be incurred by high and upper-middle-income countries, including Iran[10].

Despite the clear indications for serious action for controlling NCDs worldwide, there is a paucity of financial measurements to be used for advocacy and planning purposes in many countries. Based on this fact and the demands from the governments of many countries for such studies, a joint program by UNDP and WHO has started working on a series of investment cases around the world in collaboration with local experts. Investment on health has been considered as a concept in the public health literature because investment case studies are thought to provide important evidence for convincing the governments and policymakers to support the implementation of NCD prevention and control programs. Achieving the SDG goal of reduction in the incidence of NCDs and mortality from these diseases by 2030 requires a number of serious actions by many countries. A number of studies on NCDs investment cases that have been carried out in different countries have shown the promising results[11–14]. And the developers of this methodology have emphasized and encouraged the application of this methodology at the country level. They argue that the country-led investment cases will lead to more accurate calculations as they can use most context specific data for example on costs, coverage level, and employment rate[11]. The country-led analysis will facilitate the context analysis, as part of the analysis, which is essential for effective implementation of the investment case analysis. Earlier studies on NCD investment cases have revealed a substantial variation on cost–benefit ratios between countries. The higher the income level the higher was this ratio. M Bertram et al. have provided some reasons for these variations which have briefly been referred in the discussion of this study[11]. Hence, the developers of the methodology for NCD investment case analysis have addressed that in the future this research should be expanded to the more representative group of countries. WHO, by freely providing the OneHealth Tool, encourages countries to assess their required investments on NCD to achieve the NCD related SDG targets. This article aims to present the findings from the economic component of the investment case study performed in Iran as a collaboration of local, the author’s institutes experts and serves to catalyze inter-sectoral efforts to control NCDs in the country.

## Methods

Investment cases include an economic component that assesses four main areas, including the economic burden incurred by countries due to NCDs, the costs of interventions to control them (selected from a set of interventions designated as “best buys” by the World Health Assembly), the impacts of these interventions in decreasing the burden of NCDs, and the cost–benefit analysis of these interventions for the countries in question (return on investment)[15, 16].

A multidisciplinary team comprised of staff from the authors institutes, the United Nations Interagency Task Force on the Prevention and Control of Non-communicable Diseases, and local experts from Iranian universities conducted different phases of the study, including data gathering, intervention selection, analysis, and manuscript preparation. Clinical interventions for cardiovascular diseases and diabetes were included in our analysis, along with policy interventions targeted at tobacco, salt consumption, and physical inactivity. A complete list of interventions is provided in Table 1. Of this list, interventions were finally chosen for the Return on Investment analysis (ROI) based on the availability of relevant data for computation of both costs and health impacts. The baseline year for our analysis was 2018.

**Table 1:** The list of interventions in the study

Interventions						
**Clinical interventions**						
CVD	Treatment for those with high absolute risk of CVD/diabetes (> 30%)		Treatment of new cases of acute myocardial infarction (AMI) with aspirin		Treatment of cases with established Ischemic Heart disease (IHD) and post MI	
Diabetes	Intensive glycemic control			Retinopathy screening and photocoagulation		
**Policy interventions**						
Tobacco	Offer to help quit tobacco use: cessation	Warn about danger: Warning labels	Warn about danger: Mass media campaign	Enforce bans on tobacco advertising	Raise taxes on tobacco	Plain packaging of tobacco products
Salt	Harness industry for reformulation		Adopt standards: Front of pack labelling		Knowledge: Education and communication	
Physical Activity	Awareness campaigns to increase physical activity					

The ROI analysis included four steps:

1. Economic burden analysis
2. Calculation of costs of clinical and policy interventions
3. Assessment of the health impacts and economic benefits of the interventions
4. Return on Investment analysis for 5- and 15-year time horizons

### Economic burden analysis

To calculate NCDs’ economic burden, we used the Cost-of-Illness analysis approach to approximate the direct, and indirect costs attributable to each of the selected NCDs, including cardiovascular diseases (CVDs), diabetes, cancer, and chronic respiratory disease. The direct costs included the value of all medical care expenditures, including diagnosis, treatment, and rehabilitation costs. Indirect costs included the costs associated with the decreases in the productivity or availability of the country’s workforce, including the costs of absenteeism, presenteeism, and mortality costs.

#### Total Direct costs

The total direct costs of NCD`s were estimated via a top-down method that used the country`s National Health Accounts (NHAs). These costs included all the public and private expenditures related to NCD spending.

#### Total indirect costs

The indirect costs were computed in four steps as follows:

The annual value in terms of economic output was computed for each full-time worker in Iran based on the Gross Domestic Product (GDP) per employed person.
2.Data on the extent to which NCDs reduce labor productivity in the economy were incorporated into the calculation from the available literature on the reduction in labor force participation rate resulting from hypertension and diabetes, the reduction in full-time hours worked owing to absenteeism, and the reduction in productivity on account of presenteeism [17].
The exact number of employed people with NCDs in Iran was determined using the data on the labor force participation rate, unemployment rate, and mortality rates.
Finally, the economic losses from premature deaths were computed based on the number of active workers who had died and would be workers who could not participate in the labor market due to NCDs. Additionally, the costs associated with absenteeism and presenteeism for surviving active workers with NCDs were ascertained. The model applied the relevant productivity figures estimated in step 2 to the relevant population determined in step 3. Thus, the figure was multiplied with the Iranian GDP per employed person to arrive at the total indirect costs associated with each NCD group.

### Calculation of costs of clinical and policy interventions

We adopted a vertical program costing approach for costing of NCDs prevention program throughout the country. Two types of costs included in this approach the ingredient based costing at delivering level and the program costing at central level were estimated for clinical interventions. Since some of the activities associated with policy level interventions carried out outside of the health sector, the cost of these policies were estimated separately.

### Clinical interventions costing

#### Ingredient based costing

We used an ingredient based method to estimate the costs of interventions at delivery level. The costs of those interventions were calculated using the OneHealth Tool (OHT), which uses built-in functionality to estimate each intervention’s costs by computing the additional number of people in need of care targeted by the respective intervention multiplied by the per capita ingredient requirements for the intervention. This is finally multiplied with each ingredient unit cost to arrive at the total costs per intervention.

#### Program costing

Indeed, the program costing is seeking to quantify the value of those activities that are used at the central level for supporting the NCD program. These are activities related to training, information, supervision, evaluation, communication, administration and general program management. The OHT uses an activity-based costing (ABC) method to estimate the program costs.

### Policy level interventions

Policy level interventions are not delivered via health system, and then the costing method used for clinical interventions is not applicable. Instead, cost components of policy interventions are estimated in the same way for the program costing, ABC. The costs associated with the policy interventions were estimated with the WHO Costing Tool for NCD Prevention and Control. The tool costs human resources, training, external meetings, mass-media campaigns and other miscellaneous equipment needed to enact policies and programs based on assumptions made by the WHO experts on the magnitude of inputs required to implement and enforce each policy at the national, regional and district levels. more information about the methodology on WHO costing available from WHO CHOICE database[18].

The annual costs for both the policy and the clinical interventions were computed for a 15-year period. To compute the costs of both policy and clinical interventions, both tools require the baseline and target coverage levels for all interventions under study. The coverage levels (baseline and target) were obtained from different surveys (STEPS, IraPEN) and deliberations with experts[19].

### Assessment of the health impacts and economic benefits of the interventions

#### Health impacts

Health impacts are estimated through three effect measures of avoided incidence, avoided mortality and Healthy Life Years (HLYs) gained. The effect sizes for these measure were generated using the most valid and reliable evidence and have been built into the OHT tool. Estimating the health impacts in the OHT involves projecting forward two scenarios – the first one in which the current implementation continues as is, and another in which interventions are scaled up as per the coverage rates. The difference between the two scenarios provides us with incremental health impacts. The *avoided incidences* are modeled as result of policy and clinical interventions. The model employs the following formula to estimate the incidence of diseases in the population of interest.

$${\mathrm{I}=\left(1-\mathrm{Cov}\left({\mathrm{t}}_{1}\right)\right)*\mathrm{P}*{\mathrm{E}}_{0}*{\mathrm{R}}^{ab}+\mathrm{Cov}\left({\mathrm{t}}_{1}\right)*\mathrm{P}*\mathrm{E}}_{0}*{\mathrm{R}}^{ab-d}$$

where, *I* is the incidence of a given disease, *Cov (t*_*1*_*)* is the coverage of the intervention for those who have a given risk factor, at time “*1*”, *P* is the prevalence of those with a given risk factor, ${E}_{0}$ is the baseline prevalence of a disease event, *R* is the relative risk of a disease event for those who have a given level of a risk factor, starting from a baseline level for the risk factor, *ab* is the average number of units above a baseline level for the risk factor, *d* is the number of units of recovery towards a baseline level for the risk factor for those exposed to the intervention. Then, the change in incidence of event with increased coverage of the intervention is:

$$\Delta \mathrm{I}=\mathrm{P}*\Delta \mathrm{Cov}*{\mathrm{E}}_{0}*{\mathrm{R}}^{\mathrm{ab}}*1-{\mathrm{R}}^{-\mathrm{d}}$$

*d* is the effect of the intervention, which removes a certain percentage of the increased risk of event for those with risk factor as result of intervention. The *avoided mortality and HLYs gained* were measured based on the defined Markov health states for each disease’s pathway that were built into the OHT tool. The model uses real value of the transition probabilities to move among health states which have been extracted from the robust context specific evidence and fed into the model. In order to calculated the HLYs the disability weights associated with each state were also integrated into the model. These weights were also based on the most robust available evidence that WHO experts have incorporated into the model.

#### Economic benefits

To estimate the economic benefits of the interventions, the expected health benefits—avoided incidence, deaths, and healthy life years gained, are translated into economic gains through modeling the value of increased labor productivity (reduced indirect cost) derived from improved health, and avoided direct treatment costs. Many of the issues surrounding the monetization of indirect, and direct costs, as mentioned above, also apply to monetizing health impacts. Estimates for the net gain in worker productivity were obtained from the literature and fed into the model[15, 16].

### Return on Investment analysis

ROI was defined as the ratio of the discounted (present) value of the benefits to the costs of the health interventions. A model developed by WHO as part of the WHO/UNDP Joint Programme on Governance for NCDs in the year 2015 was used for our analysis. The tool helped us arrive at the estimates for economic gains expected to accrue from investing in both clinical and policy interventions using outputs generated by the OHT and the NCD costing tool as described above[20].

The ROI for each intervention package was arrived at by comparing the impact in terms of gains in GDP of the intervention package with the total costs of setting up and implementing the interventions using the net present value approach to future costs and economic gains with 5.8% discounting.

### Sensitivity analysis

We used a probabilistic approach to analyze the uncertainties regarding our ROI analysis results. Bootstraps of size 1000 each were created for the total costs and benefits of each intervention package. Then, we calculated ROIs for each row in each bootstrap and reported the medians, 2.5^th^ and 97.5^th^ percentiles for the resultant ROIs. Total costs and benefits were calculated by element-wise summation of the costs and benefits across all intervention group bootstraps. Then, 1000 ROIs were calculated using these sums for each of 5- and 15-year periods and the medians, 2.5^th^, and 97.5.^th^ percentiles for the resultant ROIs were reported. To build our bootstraps, we used gamma distributions with shape parameters (κ > 0) and scale parameters (θ > 0) calculated using the following equations

$$\upkappa =\frac{\overline{\mathrm{x}}}{\uptheta }$$

$$\uptheta =\frac{{\mathrm{s}}^{2}}{\overline{\mathrm{x}} }$$

where the sample mean,$\overline{\mathrm{x} }$, and the sample standard deviation, s.

## Results

### Economic burden

**Fig. 1:**
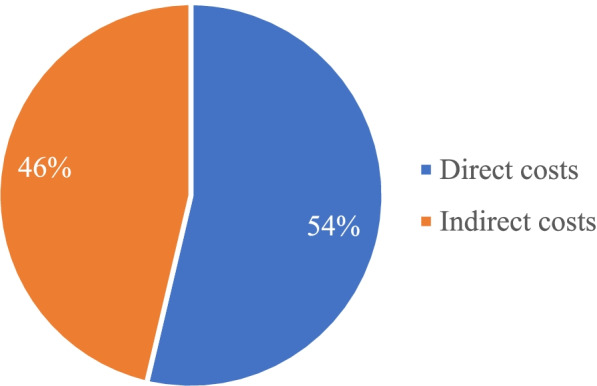
Structure of the economic burden of NCDs in Iran, 2018. The shares of direct and indirect costs of four NCD groups was estimated. Direct cost represent the highest percentage (51) in health expenditure in Iran

Figure 1 is a summary of the shares of direct, and indirect costs. Direct costs: We estimated the current health expenditure in the country to be IRR 1,240.638 trillion. Out of this expenditure, we estimated the share of four NCD groups in our study to be IRR 370.95 trillion (29.90%).

**Fig. 2:**
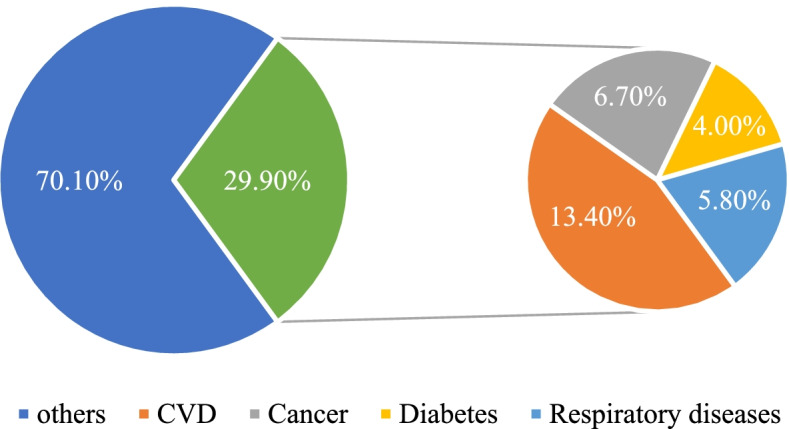
Shares of each NCD group from total health expenditure in the country Indirect costs was estimated for CVDs and Diabetes

Figure 2 summarizes the shares of each disease group from this amount.

Indirect costs: The costs of both absenteeism and presenteeism could only be computed for CVDs and Diabetes. Productivity losses resulting from absenteeism were estimated to be equivalent to a full-time productivity loss of 24.530 workers for CVD and 3.432 workers for diabetes, resulting in a total cost of absenteeism of IRR 17.71 trillion and constituting 4 percent of total indirect costs. The productivity loss due to presenteeism was equal to the full-time productivity loss of 160.96 workers for CVD and 115.11 workers for diabetes, resulting in a total burden of IRR 174.85 trillion constituting 41 percent of total indirect costs. The total costs of premature deaths were estimated to be IRR 238.17 trillion, amounting to 55 percent of all indirect costs. A detailed account of the costs attributable to each category and each disease group is presented in Table 2.

**Table 2:** Economic burden of NCDs in Iran in IRR trillions, 2018

Cost	CVD	Cancer	Diabetes	Respiratory diseases	Total
Direct costs					
Total healthcare expenditures	174.54	87.27	52.05	75.45	389.31
Indirect costs					
Cost of absenteeism	18.3	-	2.986	-	23.86
Cost of presenteeism	104.7	-	73.726	-	181
Cost of premature death	137.41	101.55	1.936	8.55	244.32
**Total indirect costs**	**260.41**	**101.55**	**77.016**	**8.55**	**449.18**
Total costs					
**Total costs**	**434.95**	**188.82**	**130.7**	**84**	**838.49**

### Intervention costs

Table 3 provides the costs of interventions in terms of net present value for the first five years and the cumulative costs for 5 and 15-year periods. Overall, clinical interventions had substantially higher costs in comparison to policy interventions. Among policy interventions, the tobacco package was the costliest.

**Table 3:** Estimated costs of policy and clinical interventions in trillion IRR, 2019–2033

Intervention type	2019	2020	2021	2022	2023	Total for 5 years	Total for 15 years
**Policy interventions**							
Tobacco	1.14	1.06	1.01	0.97	0.92	5.10	12.52
Physical activity	0.15	0.22	0.19	0.18	0.17	0.93	2.57
Salt	0.28	0.43	0.36	0.34	0.32	1.74	4.22
Total for policy interventions	1.58	1.71	1.56	1.49	1.42	7.77	19.31
**Clinical interventions**							
CVD and Diabetes	24.32	26.02	27.60	29.06	30.41	137.42	490.79
Total costs for policy and clinical interventions	25.89	27.74	29.17	30.56	31.83	145.19	511.10

### Health impacts

All interventions were estimated to lead to significant health gains in terms of healthy life years gained and mortalities averted (Table 4). Tobacco interventions were estimated to lead to the highest amounts of gain.

**Table 4:** Estimated health benefits over 15 years

Intervention package	Healthy life years gained	Mortality averted
**Policy interventions**		
Tobacco	929 097	176 071
Salt	468 875	122 750
Physical activity	468 875	122 750
**Clinical interventions**		
CVD and Diabetes	504 991	127 854

### Economic benefits

Overall economic benefits for five- and 15-year time periods as a sum of avoided direct, and indirect, costs are presented in Table 5.

**Table 5:** Costs, benefits and ROIs at five and 15 years, by intervention package (trillion IRR)

	**5 years**			**15 years**		
Intervention package	Total costs^a^	Total benefits^a^	ROI	Total costs^a^	Total benefits^a^	ROI
**Policy interventions**						
Tobacco	5.10	45.03	8.83	12.52	176.48	14.09
Physical inactivity	0.93	31.13	33.47	2.57	119.68	46.56
Salt	1.74	31.13	17.89	4.22	119.68	28.36
**Clinical interventions**						
CVD and Diabetes	137.42	31.55	.23	490.79	126.36	0.26
Total	145.17	138.86	0.95	510.1	542.22	1.06
**The results for sensitivity analysis of the ROIs**						
	**5 years**			**15 years**		
Intervention package	Median ROI (2.5^th^ and 97.5^th^ percentiles)			Median ROI (2.5^th^ and 97.5^th^ percentiles)		
Tobacco	8.79 (3.43—23.40)			14.12 (5.36—36.71)		
Physical inactivity	33.44 (12.44—91.30)			47.89 (17.75—128.32)		
Salt	18.18 (6.72—46.46)			28.71 (10.72—71.34)		
**Clinical interventions**						
CVD and Diabetes	0.23 (0.09—0.58)			0.26 (0.10—0.64)		
Total	0.99 (0.49—2.08)			1.09 (0.57—2.18)		

^a^Including direct, and indirect, costs or benefits

Combined productivity gains from both clinical and policy intervention packages in terms of net present value were calculated at IRR 230.48 trillion (roughly 1.56% of Iran’s GDP in 2017) over 15 years. Out of the productivity gains, reduced mortality (91.10%), presenteeism (4.69%), and absenteeism (4.21%) were estimated to lead to the highest economic gains, respectively.

### ROI assessment

A comparison of the costs of implementing and scaling up policy interventions with the economic benefits resulting from them demonstrated that the benefits outweigh the costs, resulting in positive ROIs both in the short (5 years) and long-run (15 years) (Table 5).

The highest ROI was observed for the physical inactivity package, followed by the package for salt interventions. The clinical interventions had ROIs well below 1, entailing their low cost-beneficence compared to the policy interventions. Bundling the clinical and policy interventions together resulted in an ROI below 1 in the 5-year period; but over the time the benefits outweighing the costs and, the resultant ROI reaches slightly above 1 for the 15-year period, signaling a possibility of a positive return on investment in the long run.

### Sensitivity analysis of the ROIs

Table 5 summarizes the results for the sensitivity analysis of the ROIs. All policy interventions had confidence intervals well above 1 for both time frames. This was while the clinical interventions had ROIs clearly below one. The results for bundling the interventions showed a possibility of ROIs both above and below 1 for both periods.

## Discussion

In this study, as the first and only NCDs investment case study in Iran, we examined the economic burden of NCDs in Iran and explored the returns on investment for four policy and clinical intervention packages selected from a set of interventions designated by the world health council as “best buys”. The investment case findings underscore the economic, social, and sustainable development toll that NCDs impose on the Iranian economy and the benefits of scaling up action.

While the investment case results confirm that Iran faces an urgent and growing NCDs epidemic, it also shows an alternate path forward. The findings show that investments in four proven and cost-effective intervention packages can significantly reduce the burden of NCDs, increasing people’s life expectancy and quality of life while decreasing the burden on the national economy. The recovered health impact and economic benefit of investing in all four policy packages would amount to 2,371,838 healthy life years gained and IRR 542.22 trillion, respectively, over a 15-year period. Increasing the productivity of human resources has always been on the agenda of the governments’ development programs in Iran. Hence, understanding the benefits that would lead to an improvement in labour productivity through investments on NCD controlling programs will lead to more supports from government officials and policymakers. On the other hand, considering the goals of SDG and UHC, the Iranian government is currently facing many challenges in achieving targets pertained to the financial protection of its citizens against medical expenses. So that, the share of out-of-pocket payments and the proportion of people facing catastrophic expenses still remain high[21, 22]. The returned money from investment on NCD can increase the financial and fiscal space of the health system to further financial protection of Iranian citizens.

Thus, these investments can contribute to the country’s overall socio-economic development, exerting positive ripple effects across society and acting as development accelerators.

The analysis drew attention to specific areas that need to be strengthened and scaled up to implement the WHO-recommended cost-effective NCD preventive and clinical interventions. Given that the packages to increase physical activity and reduce salt consumption provide the greatest returns on investment, scaling up awareness campaigns to increase physical activity and promoting healthy diets to reduce salt consumption should be given priority. Scaling up CVD and diabetes clinical interventions should not be neglected either, as the introduction of these packages could avert 127 854 deaths and lead to significant amounts of returns to Iran’s economy over a 15-year period.

Our results, to a great extent, were in line with the results from similar studies in different countries in some respects; however, we saw differences in some others. Results from other studies also revealed a substantial variation in cost–benefit ratios between countries with different income levels. M Bertram et al. argue that this condition stems from the application of context-specific factors including the way of valuing the gained health impacts as they are valued using the country-specific GDP per capita[11]. The investment case studies in Jamaica, Barbados, and Kyrgyzstan were also consistent with the results of our study in terms of finding substantially high returns on investment for tobacco use reduction programs, especially in the long run [12, 23]. The study in Kyrgyzstan also found high ROIs for salt and physical inactivity reduction programs. An essential difference between our and the above-mentioned studies’ results were the high ROIs our study yielded for the 5-year period, in contrast to these studies, which expected a more extended timeframe to reach the high ROIs. This may signify the urgency of Iran’s situation regarding these interventions and the higher potential for short-term benefits in these regards. Our results for the CVD clinical interventions packages were in line with the results from the Kyrgyzstan study, even though yielding low ROIs but remarkable economic gains for these interventions[23], highlighting the need for strategic actions to be taken to improve the efficiency in service delivery process. The cost for providing the clinical set of interventions is estimated to be high and the public finances are needed to be in place to support these interventions.

The authors recommend several steps the government can take to strengthen NCD prevention and control:

1. Raise awareness of the true costs of NCDs and the enormous development benefits of investing in the four packages of proven, cost-effective interventions among all stakeholders across the country. Doing so will strengthen public and political support for NCD prevention and control.
2. The tobacco control measures have shown a notable return on investment for Iranian context. While the government of Iran is committed to fully implement the WHO Framework Convention on Tobacco Control (WHO FCTC), and Iran’s 2015 tobacco control law is a strong piece of legislation that protects the Iranian population, but, according to interviews with experts from Ministry of health, the intensity of recommended interventions for tobacco control at country level is at low level of recommended standards. Therefore, the government could further increase the benefits of tobacco control measures by increasing the intensity of interventions.
3. Adopt a comprehensive set of salt reduction policies, regulations, and interventions. As the salt intake among Iranian population is much higher than the recommended levels[24], and on the other hand, investing on the salt reduction related interventions revealed a good return on investment for Iranian context then the government can adopt all the interventions that were explored in this investment analysis to lower the salt reduction.
4. Promote physical activity through national-level, mass public awareness campaigns, and increase leadership to ensure health is central to urban planning. Since the widespread Insufficient Physical Activity (IPA) among the Iranian adult population is of major concern[25]. In addition to mass media campaigns and physical activity initiatives, the government should strengthen multi-sectoral action to incorporate healthy/age-friendly urban development principles.
5. To improve the efficiency of service delivering methods in the country. Considering the low ROI for explored clinical interventions in comparison with the ROIs, for same interventions and with almost similar assumptions of impact in the other countries the choice of improving the efficiency needs to be given high priority.

The limitations of our study include the following: For some parameters the underlying data were taken from high-income countries as proxies that might be different from the Iranian context as lower middle income country.

## Conclusion

The results of this study underscore an essential need for the implementation of well-organized and provident policies to control the financial burdens of the NCDs in the future. The implementation of such policies, like the ones we have studied, has the potential of creating substantial improvements in the country for both the health of the citizens and the sustainability of the economy.

## Data Availability

The tens of different parameters were used in this study. The utilized data for this study were mainly the secondary data which were collected from different sources including the databases and repositories from the ministry of health of Iran, the WHO, World Bank, data in built-in tool, and the literature. The links for those data are available from: Iran_2011_STEPS_FactSheet.pdf (who.int), Avenir Health, NCD investment case guidance note final Jan 2019.cdr (who.int), Islamic Rep. | Data (worldbank.org), and some other parts of data obtained from MoH`s internal databases available from the corresponding author on reasonable request.

## Abbreviations

ROI: Return on Investment
NCDs: Non-communicable diseases
LMICs: Low- and middle-income countries
CVDs: Cardiovascular diseases
NHAs: National Health Accounts
GDP: Gross domestic product
OHT: OneHealth Tool
ABC: Activity-based costing
WHO FCTC: WHO Framework Convention on Tobacco Control
